# Cell-Specific Type I IFN Signatures in Autoimmunity and Viral Infection: What Makes the Difference?

**DOI:** 10.1371/journal.pone.0083776

**Published:** 2013-12-31

**Authors:** Chieko Kyogoku, Biljana Smiljanovic, Joachim R. Grün, Robert Biesen, Ursula Schulte-Wrede, Thomas Häupl, Falk Hiepe, Tobias Alexander, Andreas Radbruch, Andreas Grützkau

**Affiliations:** 1 German Rheumatism Research Centre (DRFZ) Berlin, An Institute of the Leibniz Association, Berlin, Germany; 2 Department of Rheumatology and Clinical Immunology, Charité University Medicine Berlin, Humboldt University of Berlin, Berlin, Germany; 3 University of Tsukuba Hospital, Amakubo, Tsukuba-shi, Ibaraki-ken, Japan; INSERM-Université Paris-Sud, France

## Abstract

Gene expression profiling of peripheral blood mononuclear cells (PBMCs) has revealed a crucial role for type I interferon (IFN) in the pathogenesis of systemic lupus erythematosus (SLE). However, it is unclear how particular leucocyte subsets contribute to the overall type I IFN signature of PBMCs and whole blood samples.Furthermore, a detailed analysis describing the differences in the IFN signature in autoimmune diseases from that observed after viral infection has not been performed to date. Therefore, in this study, the transcriptional responses in peripheral T helper cells (CD4^+^) and monocyte subsets (CD16^−^ inflammatory and CD16^+^ resident monocytes) isolated from patients with SLE, healthy donors (ND) immunised with the yellow fever vaccine YFV-17Dand untreated controls were compared by global gene expression profiling.It was striking that all of the transcripts that were regulated in response to viral exposure were also found to be differentially regulated in SLE, albeit with markedly lower fold-change values. In addition to this common IFN signature, a pathogenic IFN-associated gene signature was detected in the CD4^+^ T cells and monocytes from the lupus patients. IL-10, IL-9 and IL-15-mediated JAK/STAT signalling was shown to be involved in the pathological amplification of IFN responses observed in SLE. Type I IFN signatures identified were successfully applied for the monitoring of interferon responses in PBMCs of an independent cohort of SLE patients and virus-infected individuals. Moreover, these cell-type specific gene signatures allowed a correct classification of PBMCs independent from their heterogenic cellular composition. In conclusion, our data show for the first time that monocytes and CD4 cells are sensitive biosensors to monitor type I interferon response signatures in autoimmunity and viral infection and how these transriptional responses are modulated in a cell- and disease-specific manner.

## Introduction

Systemic lupus erythematosus (SLE) is a chronic-inflammatory autoimmune disease that affects multiple organs and is characterised by the production of autoantibodies to nuclear antigens and immune complex formation. Type I interferon (IFN) has been implicated in the development of SLE over the past 30 years [1], as elevated levels of IFN-α were detected in the serum of patients with SLE as early as 1979 [2]. Previous results from microarray studies that investigated the gene expression profiles of peripheral blood mononuclear cells (PBMCs) from patients with SLE have consistently shown an upregulation of IFN-inducible genes, such as *IFI44*, *IFI44L*, *ISG15*, *RSAD2*, *IFIT1*, *IFIT3*, *OAS1*, *OAS2*, *OASL*, *MX1*, *STAT1* and *LY6E*, when compared with healthy donors (ND).

The differential expression of IFN-inducible genes is now known as an “IFN signature” and can be used to distinguish the transcriptomes of SLE patients from ND [3]. Therefore, this signature is of potential interest for use as a surrogate IFN biomarker in diagnostic applications. Thus, the adhesion molecule SIGLEC-1 [4] and the chemokine IP-10 [5], [6] have been described as surrogate type I IFN molecules whose expression correlates with disease activity in SLE. Many studies have demonstrated type I IFN signatures in peripheral blood samples; however, different gene patterns have been suggested depending on the origin of the cells from which the analysed mRNA had been isolated. PBMCs and whole blood samples are generally used because they are easily accessible, but they also show huge variations in cellular composition, especially under chronic inflammatory conditions. Therefore, an exact allocation of genes to the appropriate cell type in which they have been differentially expressed is not possible, and a functional interpretation of these data is only possible in a limited manner.

In the field of rheumatic diseases, numerous transcriptomic studies investigating heterogeneous inflamed tissues, whole blood samples or PBMCs have been reported [7]. However, knowledge of cell type-specific transcriptional imprints in patients with SLE is lacking, and this topic has only been addressed in a very limited number of publications. To date, gene expression profiling using purified cell subsets from patients with SLE have been reported for CD4^+^ T cells [8], [9], [10], [44] and monocytes [10], [11], [44]. However, these studies were aimed at identifying a common signature that is sufficiently robust to identify the IFN responses in autoimmunity rather than to clarify how the IFN responses are modulated in a cell-specific manner.

To our knowledge, until now, only two studies have been published that compared the transcriptomes of different leucocyte subsets isolated from patients with SLE, including CD4^+^ T cells, CD8^+^ T cells, B cells, monocytes and neutrophils [10], [44]. According to the study of Lyons et al. [10], cell-specific analysis revealed more detailed information than analyses of heterogeneously composed PBMCs and showed that more than 80% of the differentially expressed genes are unique for a particular cell subset. Considering the particular functional role exerted by different leucocyte subsets in inflammation, it can be assumed that the IFN responses are modulated in a cell-specific manner. Thus, monocytes are primarily responsible for the clearance of apoptotic material, and CD4^+^ T cells, together with B cells, induce auto-reactive responses, such as autoantibody production and immune complex formation, in SLE. Although tissues such as the joints in rheumatoid arthritis (RA) and the kidneys in SLE are the major sites of inflammation, systemic effects are also detectable at the level of peripheral leucocytes [10], [11], [44]. Therefore, in this study, peripheral CD4^+^ T cells and monocytes were analysed for their cell type-specific gene expression profiles as representatives of the innate and adaptive immune system.

Although type I IFN responses are well known to be induced during viral and bacterial infections [12], [13], a direct comparison of the IFN signatures in SLE and during infection is lacking, as only two studies have been published on this topic so far. When the transcriptomes of ND immunised with the influenza vaccine and those of patients with SLE, RA, multiple sclerosis (MS) or type I diabetes (IDDM) were compared, all autoimmune individuals were found to share a common gene expression profile, but this profile was quite different from that resulting from the normal immune responses of ND following immunisation [14], [15]. Although IFN signatures are detected for almost any inflammatory response, the overall pattern of transcriptional changes and their magnitudes differs in patients with SLE from individuals infected with pathogens, such as group A streptococcus or staphylococcus [16]. To date, the molecular mechanism by which IFN responses are dysregulated in autoimmunity and whether this dysregulation is the primary cause or the consequence of the disease remains unknown.

The goal of our study was to compare the cell-specific type I IFN response signatures in SLE and viral infection by global gene expression profiling. We focused on the contribution of CD4^+^ T cells, CD16^−^ monocytes and CD16^+^ monocytes to the IFN signature observed in patients with SLE and compared these results to the pure virus-induced signatures detected in healthy individuals immunised with the yellow fever vaccine (YFV). Through this analysis, we identified genes that are involved in the chronification of IFN responses in patients with SLE.

## Results

### Detection of cell-specific IFN signatures in SLE and viral infection

**Figure 1:**
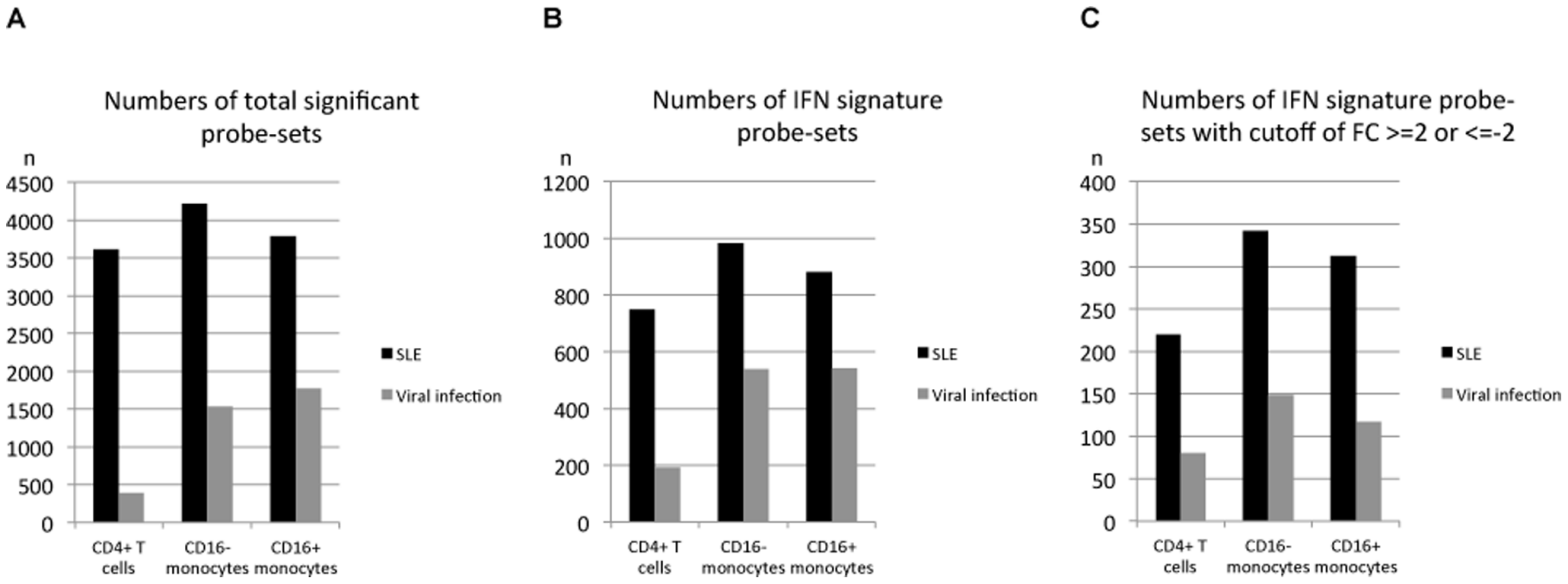
Number of probe-sets that are differentially expressed in various cell types from SLE patients and immunised healthy donors. Healthy donors (ND) immunised with the yellow fever vaccine are designated as “Viral infection”. (A) Total number of significantly differentially expressed probe-sets. (B) Number of significantly differentially expressed probe-sets from a reference list of 2442 IFN-related genes. (C) Number of significantly differentially expressed probe-sets from (B) with the additional cutoff of fold change (FC; ≥2 or ≤−2).

To discriminate between the type I IFN responses induced in active SLE patients from those induced in ND after viral infection, we compared the gene expression profiles of sorted peripheral blood CD4^+^ T cells, CD16^−^ inflammatory monocytes and CD16^+^ resident monocytes from 8 SLE patients and 4 ND 7 days after yellow fever vaccination. Upon analysing the complete list of differentially expressed genes, it was obvious that SLE resulted a much more complex immune response in both the CD4^+^ T cells (3610 probe-sets) and the monocyte subsets (4222 probe-sets in CD16^−^ and 3785 in CD16^+^ monocytes) when compared to the pure virus-induced gene pattern (Table 1 and Figure 1A). Seven days after immunisation, the virus-induced CD4^+^ T cell signature included only approximately 10% of the probe-sets identified in SLE (393 probe-sets). In contrast, the virus-induced CD16^−^ and CD16^+^ monocytes responded with 36% and 47% of the probe-sets identified in SLE, respectively (1534 and 1775 probe-sets) (Table 1 and Figure 1A).

**Table 1:** Number of probe-sets differentially expressed in SLE patients and immunized healthy donors with yellow fever vaccine.

Experimental group	Baseline group	Cell type	Total significant probes	IFN signature probes^a^	IFN signature probes (FC^b^ ≥2 or ≤−2)
SLE (n = 6)	ND before immunization^c^ (n = 4)	CD4^+^ T cells	3610	748 (20%)	220
Immunized ND^d^ (n = 4)	ND before immunization (n = 4)	CD4^+^ T cells	393	191 (49%)	80
					
SLE (n = 4)	ND before immunization (n = 4)	CD16^−^ monocytes	4222	982 (23%)	342
Immunized ND (n = 4)	ND before immunization (n = 4)	CD16^−^ monocytes	1534	540 (35%)	149
					
SLE (n = 4)	ND before immunization (n = 3)	CD16^+^ monocytes	3785	881 (23%)	312
Immunized ND (n = 3)	ND before immunization (n = 3)	CD16^+^ monocytes	1775	542 (31%)	117

This table summarizes differentially expressed probe sets as obtained by comparing arrays of the experimental group versus baseline group. Indicated are the total number of differentially expressed probe sets, the number of overlapping IFN-associated genes (absolute number and percentage of total number of differentially expressed probe sets) and the number of IFN-associated genes with fold changes ≥2 or ≤−2.

^a^ 2.442 interferon (IFN) signature genes were extracted from previous publications by Romos PS et al. [17] and Smiljanovic B et al. [11].

^b^ FC: fold change.

^c^ Expression data of healthy donors (ND) before immunization with yellow fever vaccine (YFV) was used as baseline for all comparisons.

^d^ ND 7 days after immunization with YFV.

To focus our analysis on those transcripts that are known to be regulated by IFN, we used a published list of 2220 IFN-related genes [17] combined with an additional 222 transcripts that were identified in monocytes stimulated in vitro for 90 min with IFN-α [11]. Out of these 2442 IFN-related genes, 748 probe-sets were identified in the CD4^+^ T cells from the SLE patients, while 982 and 881 probe-sets were identified in the CD16^−^ and CD16^+^ monocyte subsets, respectively (Table 1 and Figure 1B). In the immunised ND, the overlap was much lower, with 191 probe-sets in the CD4^+^ T cells and 540 and 542 probe-sets in the CD16^−^ and CD16^+^ monocyte subsets, respectively (Table 1 and Figure 1B). When we compared the ratio of the absolute number of IFN signature probe-sets to that of the total number of significant probe-sets, larger percentages of IFN-related probe-sets were observed in the immunised ND (48.6% in the CD4^+^ T cells, 35.2% in the CD16^−^ monocytes and 30.5% in the CD16^+^ monocytes) than in the patients with SLE (20.7%, 23.3% and 23.3%, respectively) (Table 1).

To narrow down our list to genes with higher fold-change (FC) values, an additional cutoff of FC ≥2 or ≤−2 was applied. Thus, 220 probe-sets in the CD4^+^ T cells from the SLE patients, 80 probe-sets in the CD4^+^ T cells from the immunised ND, 342 probe-sets in the CD16^−^ monocytes from the SLE patients, 149 probe-sets in the CD16^−^ monocytes from the immunised ND, 312 probe-sets in the CD16^+^ monocytes from the SLE patients and 117 probe-sets in the CD16^+^ monocytes from the immunised ND were identified as the top transcripts associated with type I IFN responses (Table 1 and Figure 1C). According to these lists of probe-sets, cell-specific differences between autoimmunity-related and virus-induced IFN signatures were detected, as described in the following paragraph.

### Discrimination between cell-specific IFN response signatures in autoimmunity and viral infection

**Figure 2:**
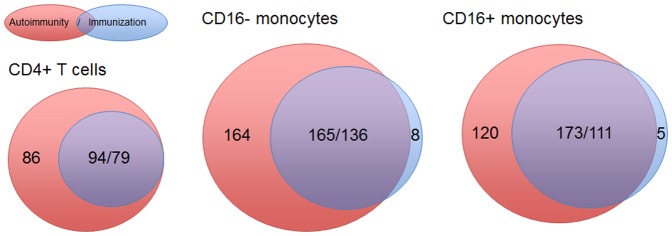
Distribution of “common” and “autoimmune-specific” IFN signature probe-sets in SLE patients and immunised healthy donors (ND). Red circles (described as “Autoimmunity”) indicate the number of IFN signature gene probes observed in the SLE patient samples. Blue circles (described as “Viral infection”) indicate the number of IFN signature gene probes observed in ND immunised with the yellow fever vaccine. The overlaps of the red and blue circles indicate “common” IFN signatures that were detected in both SLE and viral infection. When genes had several probe-sets that categorised them into multiple groups, they were excluded from the “autoimmune-/immunisation-specific” groups and only included in the “common” group. There were 11/1 (SLE/immunised ND) of these probe sets in the CD4^+^ T cells, 13/5 in the CD16^−^ monocytes and 19/1 in the CD16^+^ monocytes. Two different numbers in the area of overlaps, for example 94/79 in CD4^+^ T cells, are shown because only probe-sets that meet the cutoff of fold-change values > = 2 or < = −2 were counted in this figure.

Figure 2 shows the distribution of differentially expressed IFN signature gene probes with a FC of ≥2 or ≤−2 in the patients with SLE (in red circles) and the ND immunised with YFV (in blue circles). In the CD4^+^ T cells, the entire IFN signature observed in the immunised ND was also expressed in the patients with SLE (94 probe-sets). Similarly, most probe-sets of the IFN signatures observed in the CD16^−^ and CD16^+^ monocytes from the immunised ND were also detected in the patients with SLE (165 and 173 probe-sets, respectively). Therefore, these probe-sets were designated as the “common” IFN signature, which was observed in both autoimmunity and viral infection (Table S1, S2 and S3).

To identify the genes that were exclusively differentially expressed in each condition, all probe-sets that were not part of the “common” IFN signature were taken into account. Thus, 86, 164 and 120 probe-sets were identified as SLE-specific in the CD4^+^ T cells, CD16^−^ monocytes and CD16^+^ monocytes, respectively, whereas only very limited numbers of differentially expressed genes were identified as immunised ND-specific, with 0, 8 and 5 probe-sets in the CD4^+^ T cells, CD16^−^ monocytes and CD16^+^ monocytes, respectively (Figure 2). These signatures were designated as “autoimmune-specific” and “immunisation-specific” IFN signatures. The top candidate genes of the “autoimmune-specific” IFN signature (FC > = 4 or < = −4) are listed in Table 2, and the complete list of “autoimmune-specific” and “immunisation-specific” IFN signature genes is found in Tables S1, S2 and S3.

**Table 2:** Top candidates of differentially expressed “autoimmune-specific” IFN signature genes in CD4^+^ T cells, CD16^−^ and CD16^+^ monocytes.

Affymetrix ID	Gene Symbol	SLE CD4^+^ T cells		SLE CD16^−^ monocytes		SLE CD16^+^ monocytes	
		FC^a^	P value^b^	F^a^	P value^b^	F^a^	P value^b^
204533_at	CXCL10	5.5	2.37E-05	NS^c^	NS	NS	NS
203603_s_at	ZEB2	4.8	4.41E-10	NS	NS	NS	NS
209498_at	CEACAM1	4.2	7.19E-07	NS	NS	NS	NS
206359_at	SOCS3	4.2	1.61E-06	4.7	1.50E-05	17.1	2.57E-09
204413_at	TRAF2	4.1	1.31E-07	NS	NS	NS	NS
205699_at	MAP2K6	−4.9	1.4E-11	NS	NS	NS	NS
235086_at	THBS1	NS	NS	14.7	9.83E-05	NS	NS
201110_s_at	THBS1	NS	NS	13.7	1.46E-04	26.3	NS
38037_at	HBEGF	NS	NS	11.8	2.55E-07	2.6	4.21E-05
208937_s_at	ID1	NS	NS	11.4	1.65E-11	NS	5.48E-03
202340_x_at	NR4A1	NS	NS	10.8	8.37E-10	2.3	NS
210397_at	DEFB1	NS	NS	10.7	4.77E-18	NS	1.08E-06
204420_at	FOSL1	NS	NS	10.3	6.28E-12	NS	NS
201109_s_at	THBS1	NS	NS	8.9	3.68E-04	NS	NS
205409_at	FOSL2	NS	NS	6.9	1.43E-13	NS	NS
205767_at	EREG	NS	NS	6.3	2.63E-05	NS	NS
203821_at	HBEGF	NS	NS	5.7	6.50E-07	2.5	NS
212285_s_at	AGRN	NS	NS	5.6	3.34E-07	NS	3.56E-03
208075_s_at	CCL7	NS	NS	5.3	1.05E-02	NS	NS
201005_at	CD9	NS	NS	4.7	1.02E-03	8.2	NS
224454_at	ETNK1	NS	NS	4.4	1.74E-14	NS	1.37E-03
218880_at	FOSL2	NS	NS	4.0	1.38E-14	3.9	NS
220088_at	C5AR1	NS	NS	3.9	8.81E-19	NS	NS
222566_at	SUV420H1	NS	NS	−4.1	8.01E-12	−3.6	6.75E-11
204798_at	MYB	NS	NS	−4.6	1.03E-04	NS	2.43E-08
230337_at	SOS1	NS	NS	−6.5	2.66E-05	−3.6	NS
209795_at	CD69	NS	NS	NS	NS	56.0	3.30E-16
210512_s_at	VEGFA	NS	NS	NS	NS	18.3	4.62E-08
200923_at	LGALS3BP	NS	NS	NS	NS	12.8	3.17E-08
227697_at	SOCS3	3.1	1.16E-06	3.7	1.35E-04	11.8	1.90E-15
210164_at	GZMB	2.0	2.12E-03	NS	NS	10.4	9.16E-08
219257_s_at	SPHK1	NS	NS	2.7	2.38E-12	9.0	1.49E-04
214617_at	PRF1	NS	NS	NS	NS	7.6	1.34E-07
220576_at	PGAP1	NS	NS	NS	NS	7.5	7.48E-05
206115_at	EGR3	NS	NS	NS	NS	6.3	3.74E-04
211828_s_at	TNIK	NS	NS	NS	NS	6.2	1.54E-03
205488_at	GZMA	NS	NS	NS	NS	6.0	1.40E-06
224516_s_at	CXXC5	NS	NS	NS	NS	4.7	1.57E-03
205863_at	S100A12	2.4	7.15E-02	NS	NS	4.7	1.74E-10
207655_s_at	BLNK	NS	NS	NS	NS	4.3	3.99E-04
213524_s_at	G0S2	NS	NS	NS	NS	4.2	1.29E-06
1569150_x_at	PDLIM7	NS	NS	2.3	3.63E-11	4.0	2.40E-06
202498_s_at	SLC2A3	NS	NS	2.1	6.17E-07	4.0	2.60E-07
228846_at	MXD1	NS	NS	NS	NS	4.0	6.23E-03
203618_at	FAIM2	NS	NS	NS	NS	−4.9	9.33E-07

This table summarizes top candidates of differentially expressed “autoimmune-specific” IFN signature genes in CD4^+^ T cells, CD16^−^ and CD16^+^ monocytes defined by a foldchange ≥4 or ≤−4.

^a^ FC: fold change.

^b^ P values was calculated by the comparison of SLE versus healthy donors before immunization. All values were Bonferroni-corrected for multiple-comparisons.

^c^ NS: Not significant with cutoff of FC > = 2 or < = −2 in SLE.

**Figure 3:**
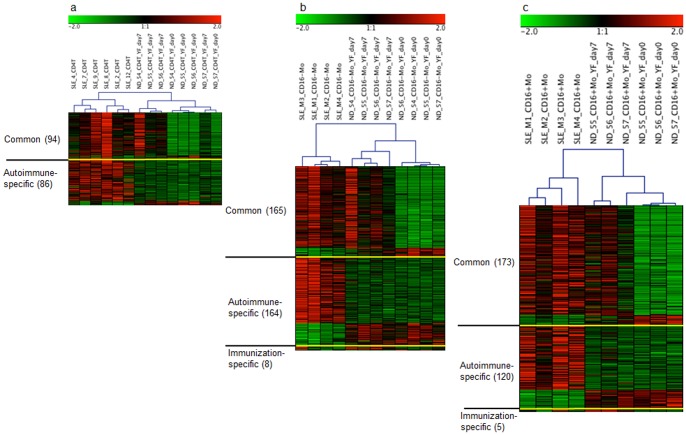
Cluster diagrams of SLE patients and healthy donors before and after immunisation with the yellow fever vaccine. Healthy donors (ND) immunised with the yellow fever vaccine are designated as “ND_YF_day7”, and ND before immunisation are designated as “ND_YF_day0”. (A) IFN signature in CD4^+^ T cells. The 94 probe-sets of “common” IFN signatures observed both in the SLE patients and immunised ND and the 86 probe-sets of “autoimmune-specific” IFN signatures observed only in the SLE patients distinguish the SLE patients from the immunised ND. (B) IFN signature in CD16^−^ monocytes. The 165 probe-sets of “common” IFN signatures, 164 probe-sets of “autoimmune-specific” IFN signatures and 8 probe-sets of “immunisation-specific” IFN signatures distinguish the SLE patients from the immunised ND. (C) IFN signature in CD16^+^ monocytes. The 173 probe-sets of “common” IFN signatures, 120 probe-sets of “autoimmune-specific” IFN signatures and 5 probe-sets of “immunisation-specific” IFN signatures distinguish the SLE patients from the immunised ND.

These “common”, “autoimmune-specific” and “immunisation-specific” IFN signature probe-sets were used for a hierarchical cluster analysis, shown in Figure 3. The determination of cell-specific signatures allowed for a classification of the SLE patients, ND and immunised ND (Figure 3A for the CD4^+^ T cells, Figure 3B for the CD16^−^ monocytes and Figure 3C for the CD16^+^ monocytes). Only one immunised ND (ND_57) was misclassified in the analyses of the CD4^+^ T cells and CD16^+^ monocytes and clustered with the ND samples before immunisation (Figure 3A and 3C).

### Cell-specific IFN signatures can be used to classify PBMCs from independent SLE and yellow fever vaccinated individuals

**Figure 4:**
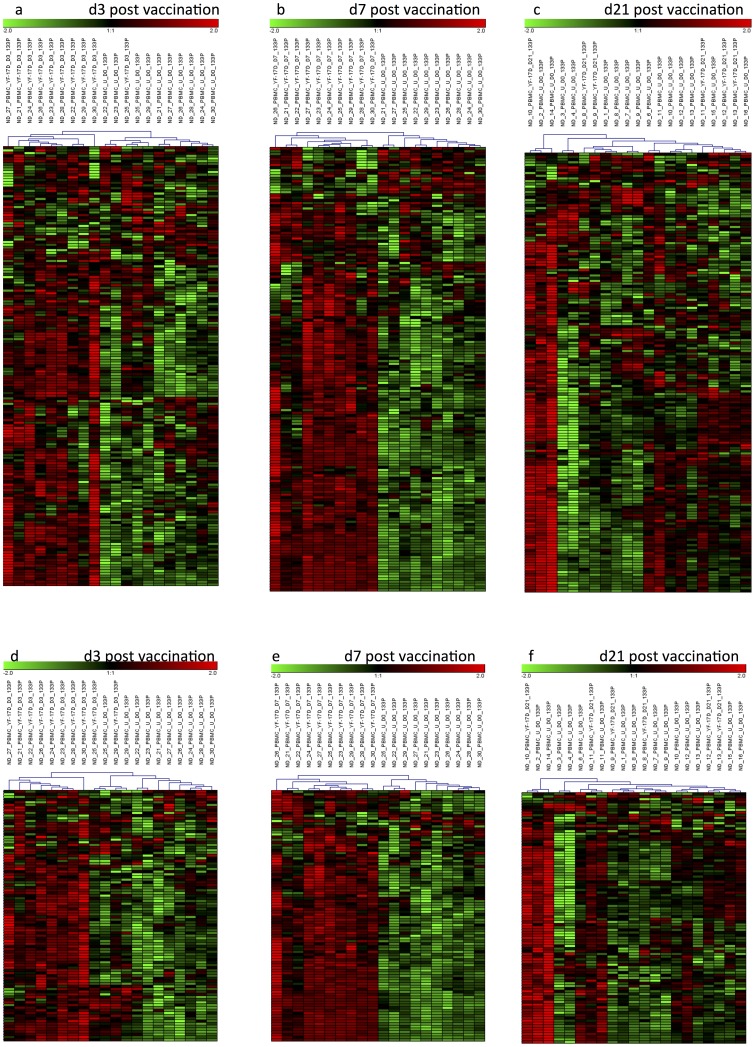
For the validation of 165 monocyte- (a, b and c) and 94 T helper lymphocyte-specific (d, e and f) common-IFN signature genes, their expression were followed in PBMC's of an independent cohort of yellow-fever vaccinated individuals. Expression profiles were generated from PBMC's at day 3 (a and d), 7 (b and e) and 21 (c and f) after vaccination and compared to baseline levels at d0. Both, the common type I interferon signatures of monocytes and CD4 lymphocytes allowed the monitoring of the induction of interferon responses in PBMCs at d3, peaking at d7 and almost declining at d21.

We used publically available gene expression data from PBMCs of juvenile SLE and PBMCs of yellow fever vaccinated individuals d3, d7 and d21 post infection to demonstrate that cell-specific interferon signatures identified in a limited set of samples are robust enough to allow a classification by hierarchical clustering. As shown in figure 4, a correct classification of samples before, 3 and 7 days after vaccination was achieved by using the CD14^+^CD16^−^- and CD4^+^- common-IFN signatures, respectively (figure 4a, b and 4d, e, respectively). Only one sample (ND_25) obviously showed a delayed immune response signature and clustered together with the sample taken before vaccination. This supposed miss-classification was observed for using the cell-specific signatures from monocytes and T helper cells. But 4 days later the vaccination response of this individual was almost comparable to the other ones. As expected, at day 21 when the type I interferon response was completely subsided a more or less random clustering of samples was observed (figure 4c). Using the autoimmune-specific gene signatures of monocytes (164 probe sets) and T helper cells (72 probe sets) allowed now classification of PBMC's 3 (figure S1a and c) and 7 days (figure S1b and d) post vaccination as shown for the common IFN signature (figure 4).

**Figure 5:**
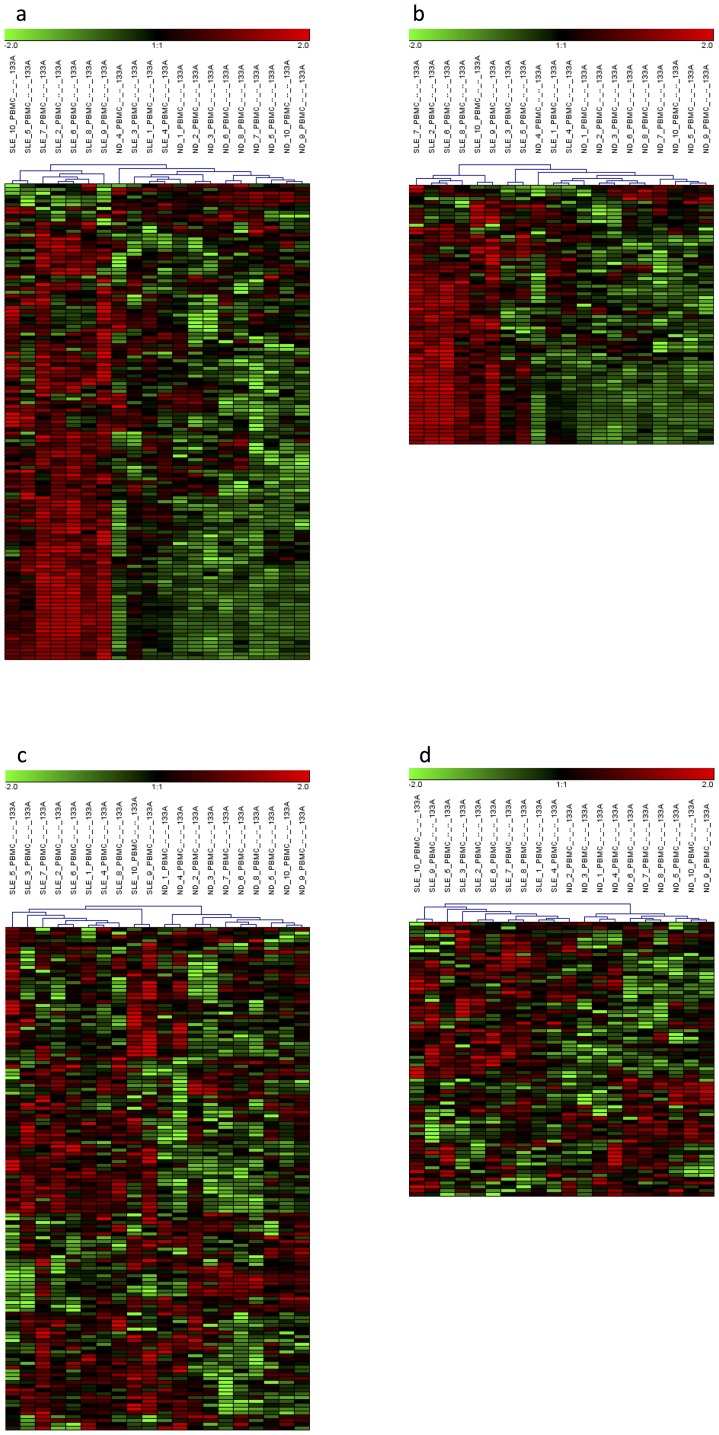
For the validation of the 125 monocyte- (a) and the 68 T helper lymphocyte-specific (b) common-IFN signature genes their expression were followed in PBMC's of an independent cohort of juvenile SLE patients. In addition, we used these samples to validate the 132 monocyte- (c) and the 72 T helper cell-specific (d) gene signatures, which have been identified as autoimmune-specific type I IFN response signatures in these cell types. The common IFN signatures of both cell types allowed a correct classification in 8 out of 10 samples. Only the autoimmune-specific IFN signature of monocytes allowed a correct grouping of all SLE samples.

Hierarchical clustering of new juvenile SLE samples by using the common-type I IFN-signature genes identified in monocytes (125 genes) allowed a correct classification of 7 out of 10 SLE samples (figure 5a). Only samples SLE_1, SLE_3 and SLE_4 were grouped within the healthy controls. This result was partly confirmed by using the corresponding type I IFN signature genes (68 genes) identified in CD4 lymphocytes (figure 5b). Here, SLE1 and SLE_4 also clustered within the healthy controls, but in addition, SLE_3 and SLE_5 formed a subcluster showing a diminished common IFN signature if compared to the other SLE-samples.

A perfect clustering result was obtained if the autoimmune-specific type I IFN signature from monocytes was applied (132 genes), although the overall gene pattern of up- and down regulated transcripts is not as striking when compared to the common signature (figure 5c). The corresponding CD4 signature (72 genes) also showed a clear separation of SLE and healthy samples (ND) with one ND sample (ND_2) clustering within the group of SLE samples (figure 5d).

The number of signature genes used for the classification of new lupus samples was reduced because these samples were hybridized on the older HG-U133A array not covering the same number of probe sets used on the latest version of the HG-U133Plus 2.0 array. Since these different array formats used different feature sizes a direct clustering of jSLE and yellow fever PBMC samples was not possible.

### Absolute expression levels of the “common” IFN signature genes are stronger in patients with SLE than in immunised ND

Table 3 shows the top candidate genes (FC ≥10 or ≤−10 in SLE) of the “common” IFN signature detected in the patients with SLE and immunised ND. Previously reported well-known IFN signature genes, such as *IFI27, LY6E*, *IFI44*, *IFI44L*, *RSAD2*, *OAS1*, *OASL*, *IFIT3* and *IFIT1*, were found to be upregulated in all of the cell types investigated. The top cell-specific genes with respect to FC were *LAMP3* in the CD4^+^ T cells (FC in SLE 11.0 and FC in immunised ND 9.0), *CCL2* in the CD16^−^ monocytes (FC in SLE 14.6 and FC in immunised ND 15.9) and *SIGLE1* in the CD16^−^ monocytes (FC in SLE 24.0 and FC in immunised ND 16.6) and the CD16^+^ monocytes (FC in SLE 165.0 and FC in immunised ND 62.5). The absolute gene expression values of these genes in the different cell types from the SLE patients and immunised ND are listed in Table S4.

**Table 3:** Top candidates of differentially expressed cell-specific “common” IFN signature genes in patients with SLE and immunized healthy donors.

Affymetrix ID	Gene Symbol	CD4^+^ T cells (FC^a^)		CD16^−^ monocytes (FC)		CD16^+^ monocytes (FC)	
		SLE^b^	Immunized ND^c^	SLE	Immunized ND	SLE	Immunized ND
216598_s_at	CCL2	NS^d^	NS	14.6	15.9	NS	NS
209795_at	CD69	NS	NS	27.3	8.7	NS	NS
219895_at	FAM70A	NS	NS	9.2	5.7	15.5	3.8
221345_at	FFAR2	NS	NS	9.8	2.3	10.3	3.6
204187_at	GMPR	NS	NS	10.2	7.4	8.5	5.9
211267_at	HESX1	NS	NS	16.0	10.0	6.4	3.3
202411_at	IFI27	47.1	5.6	37.6	19.4	33.5	15.2
214059_at	IFI44	3.9	5.3	6.9	6.8	10.6	6.3
204439_at	IFI44L	17.7	16.4	13.3	11.2	22.1	17.7
203153_at	IFIT1	14.7	16.1	9.8	10.7	10.2	11.5
204747_at	IFIT3	10.4	12.0	6.1	7.7	4.4	5.6
229450_at	IFIT3	18.1	22.4	6.2	7.2	3.6	4.2
201601_x_at	IFITM1	NS	NS	15.8	9.9	5.3	4.7
214022_s_at	IFITM1	NS	NS	12.2	10.7	5.0	4.2
205569_at	LAMP3	11.0	9.0	NS	NS	NS	NS
200923_at	LGALS3BP	NS	NS	10.9	5.0	NS	NS
226702_at	LOC129607	11.7	9.7	7.0	5.7	6.6	4.7
202145_at	LY6E	20.3	5.0	27.5	8.3	10.3	4.0
202869_at	OAS1	11.4	9.2	3.0	2.9	2.3	2.0
205552_s_at	OAS1	15.1	9.5	3.3	3.0	2.1	2.2
205660_at	OASL	6.2	4.1	11.7	6.9	13.7	8.4
213797_at	RSAD2	12.8	13.2	8.0	11.4	11.3	13.7
242625_at	RSAD2	15.0	12.8	13.4	12.7	14.3	14.3
200986_at	SERPING1	NS	NS	10.2	10.3	15.4	8.6
219519_s_at	SIGLEC1	NS	NS	24.0	16.6	165.0	62.5
44673_at	SIGLEC1	NS	NS	18.7	14.0	26.9	13.2

This table summarizes top candidates of differentially expressed cell-specific “common” IFN signature genes in patients with SLE and immunized healthy donors defined by a fold change ≥10 or ≤−10 in SLE.

^a^ FC: fold change.

^b^ Fold change (FC) by the comparison of SLE versus healthy donors (ND) before immunization.

^c^ FC by the comparison of ND 7 days after immunization with yellow fever vaccine versus ND before immunization.

^d^ NS: Not significant with cutoff of FC > = 2 or < = −2 in SLE.

Comparing the relative strength of the IFN responses of the top ranked “common” IFN signature probes, it was obvious that the expression levels of the “common” IFN signature genes were relatively stronger in the patients with SLE than in the immunised ND (Table 3). For example, a comparison of the FCs for *IFI27*, *LY6E* and *IFI44L* is shown in Figure S2A. Considering all of the “common” IFN signature genes (FC ≥2 or ≤−2 in SLE), 76 of 94 “common” IFN signature probes (81%) in the CD4^+^ T cells (Table S1), 120 of 165 “common” IFN signature probes (73%) in the CD16^−^ monocytes (Table S2) and 145 of 173 “common” IFN signature probes (84%) in the CD16^+^ monocytes (Table S3) showed higher expression levels with larger FCs in the SLE patients than in the immunised ND. The average FC of all of the “common” IFN signature gene probe-sets in SLE was higher than that in viral infection for all cell types examined (Figure S2B). This trend is also clearly visible in the cluster diagrams of Figure 3A, 3B and 3C.

### Functional annotation analysis of the “autoimmune-specific” and “common” IFN signatures for autoimmunity and viral infection

To evaluate the functional role of the genes identified as “common” and “autoimmune-specific” IFN signatures, we performed Ingenuity Pathway Analysis (IPA). For IPA, we used the complete list of significantly differentially expressed IFN signature genes without a FC cutoff (Table 1).

**Figure 6:**
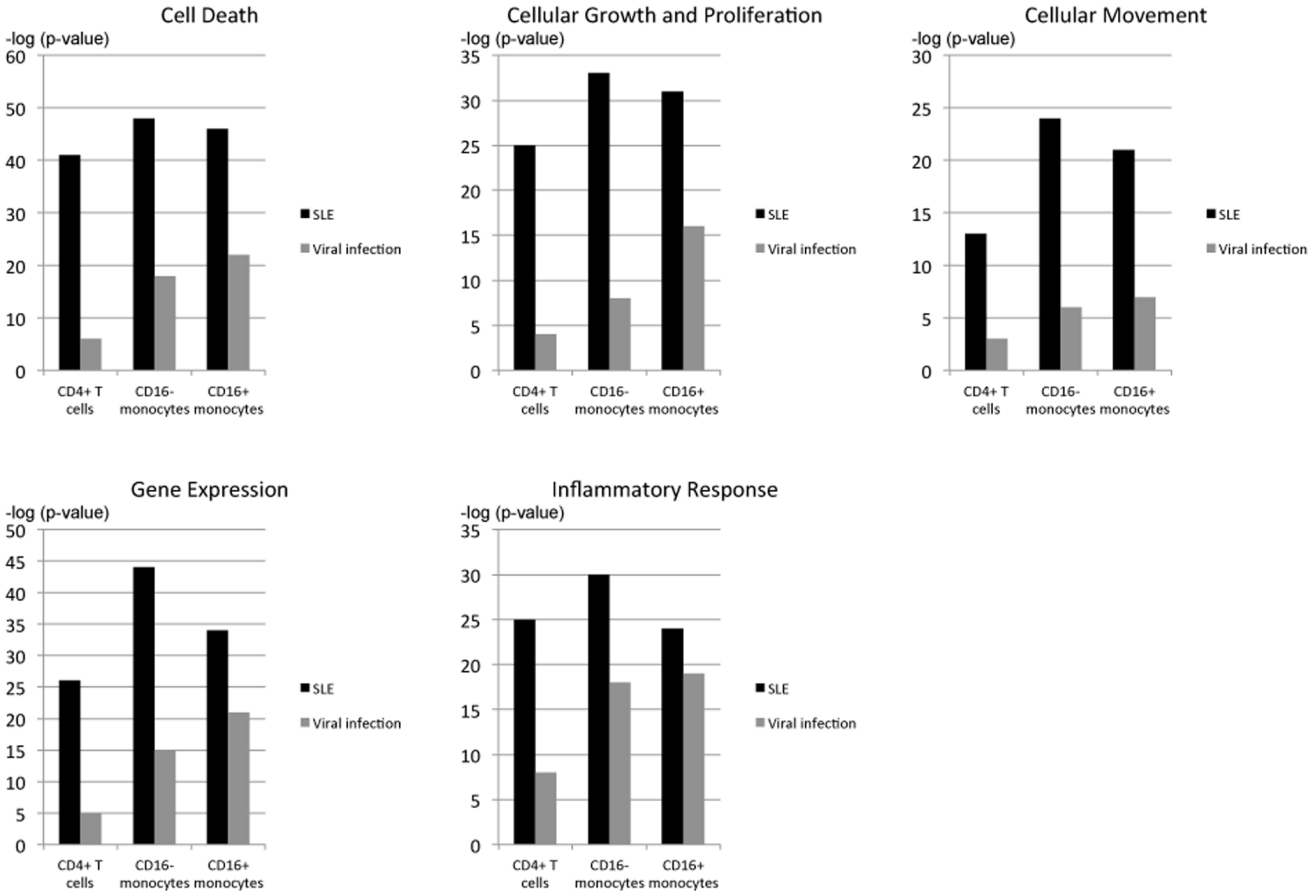
Comparison of the enrichments of genes with selected biological functions in SLE patients and immunised healthy donors. CD4^+^ T cells, CD16^−^ monocytes and CD16^+^ monocytes from patients with SLE and healthy donors immunised with the yellow fever vaccine (designated as “Viral infection”) were analysed using the Ingenuity Pathway Analysis (IPA) tool.

In Figure 6 and Table S5, the basic biological functions of immune cells, such as “cell death”, “cellular growth and proliferation”, “cellular movement”, “gene expression” and “inflammatory response”, were compared. The cell-specific IFN signature identified for SLE showed a higher significance for all biological functions considered in the IPA compared to that identified for viral infection. In addition, the turnover of cells, which is regulated by apoptosis and proliferation, was a significant biological function, suggesting that pro-apoptotic events occur in SLE.

**Figure 7:**
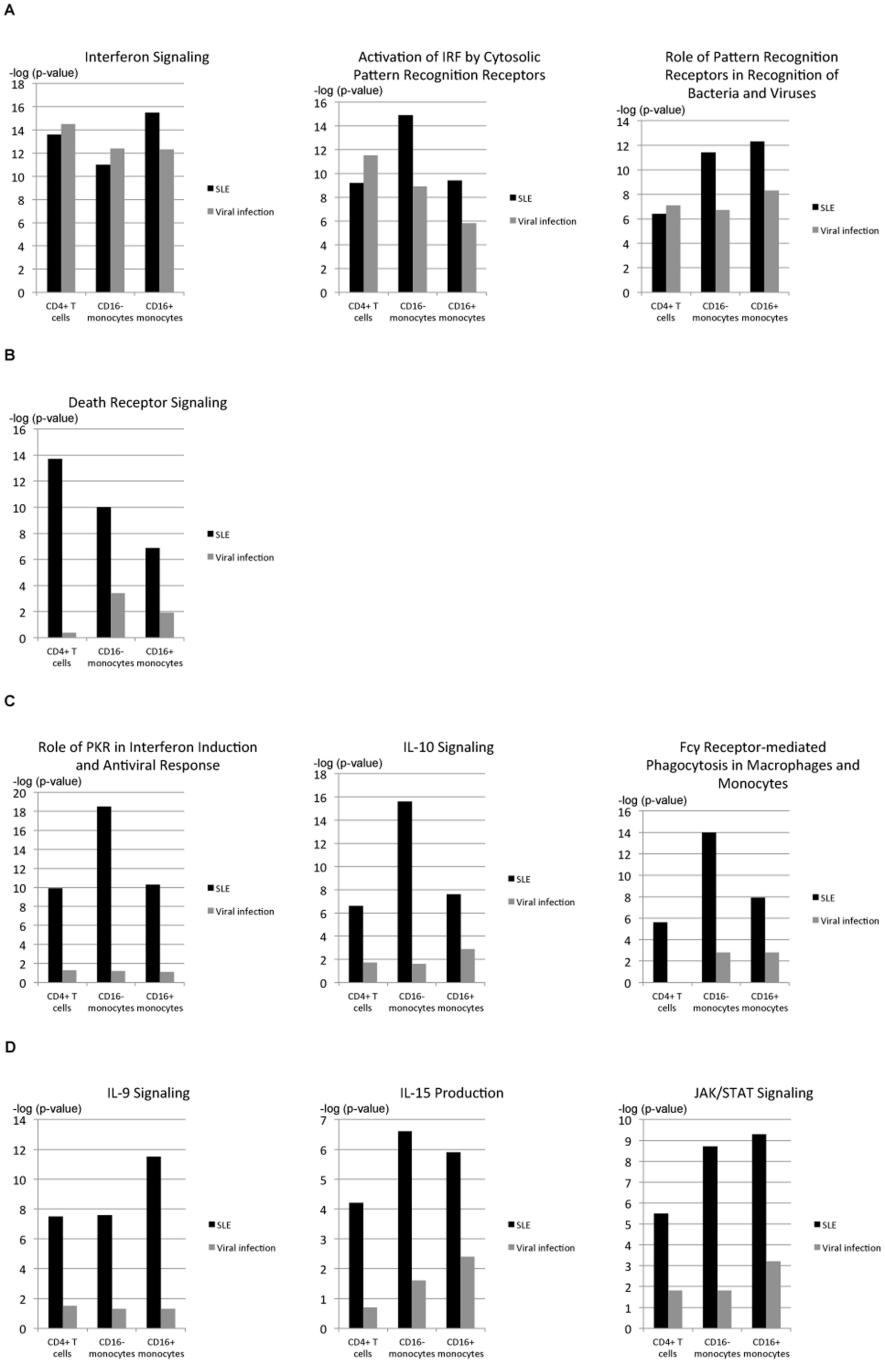
Comparison of the enrichments of genes in the top-ranked canonical pathways in SLE patients and immunised healthy donors. CD4^+^ T cells, CD16^−^ monocytes and CD16^+^ monocytes from patients with SLE and healthy donors immunised with the yellow fever vaccine (designated as “Viral infection”) were analysed using the Ingenuity Pathway Analysis (IPA) tool. (A) Commonly observed pathways in all compared groups. (B) Dominantly observed pathways in the CD4^+^ T cells from the SLE patients. (C) Dominantly observed pathways in the CD16^−^ monocytes from the SLE patients. (D) Dominantly observed pathways in the CD16^+^ monocytes from the SLE patients.

When examining the canonical pathways, “interferon signalling”, “activation of IRF by cytosolic pattern recognition receptors” and “role of pattern recognition receptors in recognition of bacteria and viruses” were ranked as top pathways that were common for all of the compared groups (Figure 7A and Table S6). Accordingly, the expression of *TLR4* (FC in CD16^−^ monocytes/CD16^+^ monocytes; FC 1.9/1.7 in SLE, FC 1.3/1.4 in immunised ND), *TLR7* (FC 1.4/2.3 in SLE, FC 1.8/1.9 in immunised ND) and *DDX58/RIG-1* (FC in CD4+ T cells/CD16^−^ monocytes/CD16^+^ monocytes; 2.1/2.6/2.8 in SLE, FC 2.1/3.4/2.7 in immunised ND) were upregulated in all cell types from the patients with SLE and immunised ND (Table S4).

The top ranked pathway found in the CD4^+^ T cells from the SLE patients was “death receptor signalling” (Figure 7B and Table S6). This was demonstrated by the upregulated expression of *FAS* in the patients with SLE (FC 2.0 in the CD4^+^ T cells, FC 1.8 in the CD16^−^ monocytes and FC 1.3 in the CD16^+^ monocytes) (Table S4).

The top ranked pathways identified in the CD16^−^ monocytes from the SLE patients were “role of PKR (protein kinase-R; serine/threonine protein kinase) in interferon induction and antiviral response”, “IL-10 signalling” and “Fcγ receptor-mediated phagocytosis in macrophages and monocytes” (Figure 7C and Table S6). As a consequence of IL-10 signalling, the expression of *SOCS3* was highly upregulated in the SLE patients (FC 4.2 in the CD4^+^ T cells, FC 4.7 in the CD16^−^ monocytes and FC 17.1 in the CD16^+^ monocytes) (Table S4). Furthermore, inflammation-related signalling pathways, such as “TNFR1/2 signalling”, “IL-1 signalling”, “IL-6 signalling” and “IL-17 signalling”, were greatly affected (Figure S3).

In the CD16^+^ monocytes from the SLE patients, the differentially expressed genes were primarily assigned to the “IL-9, IL-15 and JAK/STAT signalling” pathways (Figure 7D and Table S6). Accordingly, the expression levels of *IL-15/IL-15RA* (FC 1.4/1.2 in the CD4^+^ T cells, FC 1.4/1.4 in the CD16^−^ monocytes and FC 1.5/1.8 in the CD16^+^ monocytes) and *IL2RG* (FC 2.8 in the CD4^+^ T cells, FC 2.9 in the CD16^−^ monocytes and FC 1.9 in the CD16^+^ monocytes) were found to be upregulated in the SLE patients (Table S4). The expression levels of pro-inflammatory cytokines that were previously reported to be efficiently produced by CD16^+^ monocytes, such as *TNF* (FC 3.3 in the CD16^−^ monocytes and FC 3.5 in the CD16^+^ monocytes) and *IL-1β* (FC 3.3 and FC 2.3, respectively), were similarly upregulated in both monocyte subsets isolated from patients with SLE (Table S4). Based on the previously suggested function of CD16^+^ resident monocytes, which preferentially translocate to tissues and efficiently function in antigen presentation, “actin cytoskeleton signalling”, “chemokine signalling” and “antigen presentation pathway” are compared in Figure S3. The relevance of these signalling pathways was increased in the CD16^−^ classical inflammatory monocytes compared to the CD16^+^ resident monocytes or was quite similar in both monocyte subsets.

## Discussion

This study is the first to demonstrate quantitative and qualitative differences between IFN signatures in autoimmunity and viral infection using purified CD4^+^ T cells and monocyte subsets. We were able to discriminate between cell-specific viral response signatures and the pathogenically amplified IFN signatures observed in autoimmunity. The differences were of both a qualitative and quantitative nature, as the signatures in the patients with SLE were characterised by much more complexly compiled gene patterns with increased absolute gene expression levels. For the identification of IFN responses in autoimmunity and viral infection, we used a reference list of 2442 IFN-related genes. Although this gene list is based on previous publications of associated genes in patients with SLE and computational searches for IFN-related genes [17] complemented by our previous experimental data obtained from monocytes stimulated for 90 minutes in vitro with IFN-α [11], it is not intended to be exhaustive. Because different cell types respond to IFN with specific gene expression signatures [11], the analysis of cell types other than CD4^+^ cells and monocytes would be helpful for a comprehensive description of type I IFN-related genes.

We are aware that our study design is based on rather limited numbers of patient samples because active SLE patients are often leucopaenic. Thus, a relatively large volume of blood (at least 50 ml) was necessary to perform the cell sorting experiments. To reduce inter-individual variation as much as possible, only clinically well-characterised and active patients were selected so that untreated patients or patients treated with maximum 10 mg prednisolone per day were included in this study. Finally, our highly validated gene-filtering algorithm [18] was optimised in such a way that the statistical analysis was performed based on pair-wise comparisons, i.e., comparing 4 versus 4 arrays, resulting in a statistical power of 16 comparisons, and provided significant results for limited numbers of samples. Considering the overall pattern of the differentially expressed probe-sets in the immunised ND, which were not restricted to only IFN-related genes, more genes were found in the monocytes than in the CD4^+^ T cells, whereas in the SLE patients, more or less comparable numbers of IFN-responding transcripts were identified. This quantitative relationship was also detectable when only IFN-associated transcripts were considered, suggesting that both subsets of monocytes show a more complex transcriptional regulation in response to IFN compared to CD4^+^ T cells in viral infection. Therefore, it can be assumed that monocytes, as the foot soldiers of the innate immune system, are the more sensitive biosensors in detecting viral infections compared to CD4^+^ T cells.

When we estimated the contribution of the IFN-associated transcripts to the overall gene signature, it was confirmed that in viral infection, type I IFNs are the major anti-viral cytokines. In SLE, type I IFNs also dominate particular cell responses in CD4^+^ cells and monocytes, as previously described [10], [19], [44], but these responses are modulated by additional immunoregulatory events. These events may be responsible for the amplified levels of the “common” and “autoimmune-specific” IFN signature genes, which reflect the chronically sustained IFN response observed in patients with SLE [20]. The “common” and “autoimmune-specific” IFN signatures clearly distinguished between patients with SLE and viral infection at the molecular level and, therefore, are of potential interest as biomarkers to enable the differentiation between SLE flares and viral infections. It can be speculated that one exceptional immunised ND who clustered with the unimmunised ND might have shown a poor response to vaccination against yellow fever, as it is known that the T cell response to YFV is variable from one individual to another, at least compared to PBMCs [13]. The robustness and reliability of cell-specific type I interferon signatures identified was demonstrated by using public data for PBMCs obtained from juvenile lupus patients [45] and from yellow fever vaccinated healthy individuals at d3, d7 and d21 after immunisation [46]. So it was possible to monitor induction and remission of type I IFN responses after vaccination and to classify successfully 70%–100% of juvenile SLE samples. Obviously, the type I interferon-driven pathologies in juvenile and adult onset of SLE are closely related with respect to the qualitative composition and the strength of the interferon response signature.

This validation shows for the first time that cell-specifically generated expression profiles can be used as classifiers in PBMCs although usually their cellular composition shows wide inter-individual variations, especially in patients with chronic inflammation. Therefore, it seems reasonable to assume that using cell-specific gene signatures for analyzing whole blood or PBMC samples results in more robust signatures as compared to signatures originally obtained directly from whole blood cells or PBMCs.

Looking at the cell-specific IFN signature in more detail, *LAMP3* (lysosomal-associated membrane protein 3) in the CD4^+^ T cells, *CCL2* (chemokine [C-C motif] ligand 2) in the CD16^−^ monocytes and *SIGLEC1* (sialic acid binding Ig-like lectin 1) in both the CD16^−^ and CD16^+^ monocytes were identified as potential cell-specific surrogate IFN markers for SLE and viral infection, whose increased absolute expression values were observed in autoimmunity.

LAMP3 is primarily located in lysosomes, shuttles between lysosomes, endosomes and the plasma membrane by exocytosis and is known to be involved in antigen presentation by dendritic cells [21]. The increased expression of *LAMP3* in the CD4^+^ T cells from the SLE patients and immunised ND may affect the TCR-antigen interaction by altering the surface expression of TCR and co-stimulatory molecules, such as CD28 and ICOS. In fact, our data detected the slightly increased expression of *CD3E* (FC 1.9), *CD3G* (FC 1.3) and *ICOS* (FC 1.6) in the CD4^+^ T cells from the SLE patients but, interestingly, not in the CD4^+^ T cells from the immunised ND.

CCL2 is primarily secreted by monocytes, macrophages and dendritic cells, and its receptor CCR2 is known as a surface marker for CD16^−^ monocytes [22]. Secreted CCL2 is tethered on endothelial cells by glycosaminoglycan (GAG) side chains of proteoglycans [23]. Thus, the CCR2-CCL2 interaction is an essential mechanism for CCR2-expressing CD16^−^ monocytes to migrate from the peripheral bloodstream into the inflamed tissues [24], [25]. The upregulated expression of *CCL2* in the CD16^−^ monocytes from the SLE patients suggests an enhanced infiltration of CD16^−^ monocytes into the tissue, although the expression of *CCR2* was slightly decreased (data not shown). This can explain the leucopaenia that is often observed in patients with SLE.

SIGLEC1 is a lectin-like adhesion molecule that binds glycolised ligands on the cell surface in a sialic acid-dependent manner. We have previously reported that the expression of SIGLEC1 in monocytes correlates with the disease activity of SLE and the levels of anti-dsDNA antibodies [19]. In this study, we confirmed that in both monocyte subsets, a striking increase in *SIGLEC1* mRNA expression was observed in patients with SLE. However, when comparing FCs, significantly higher values were detected for the CD16^+^ monocytes (FC 165.0), which was caused by a near absence of transcripts in the ND (the mean signal intensity in ND was 9.4) (Table S4), whereas the CD16^−^ monocytes showed a higher and more heterogeneous basal expression (FC of 24.0, the mean signal intensity in ND was 92.3) (Table S4). According to these data, CD16^+^ resident monocytes would be the more sensitive biosensors to monitor *SIGLEC1* expression in patients with SLE. However, unfortunately, these cells are only rarely detected and are completely absent in a subgroup of severely leucopaenic SLE patients.

According to our computational analysis of biological function by IPA, it was suggested that cells from patients with SLE have an increased turnover and are characterised by a hyper-apoptotic status. This was further supported by our analysis of the canonical pathways, which showed that Fas-mediated apoptosis predominantly increased in the CD4^+^ T cells from the SLE patients. It is known that the immune cells of patients with SLE show increased pro-apoptotic behaviour, which is accompanied by decreased clearance of apoptotic debris. This ultimately facilitates the enhanced formation of immune complexes, which is a typical histopathological hallmark of SLE [1].

Using an IPA search for the top-ranked canonical pathways that are commonly responsible for autoimmunity and viral infection, “pattern recognition”-related genes, such as toll-like receptor (*TLR*), both expressed on the cell surface (*TLR4*) and intracellularly (*TLR7*), and *DDX58* (DEAD [Asp-Glu-Ala-Asp] box polypeptide 58, alternatively called *RIG-1*) [26] were enriched in all cell types from patients with SLE and immunised ND, suggesting the existence of former viral responses in the disease history of the SLE patients. Our data support the hypothesis that the dysregulated and most likely sustained anti-microbial innate immune response mediated by TLR4- and TLR7-receptor signalling is a major mechanism involved in the pathogenesis of SLE.

In the CD16^−^ monocytes from the SLE patients, gene enrichment in “IL-10 signalling” was found to be dominant by IPA. IL-10-mediated STAT3 activation is known to induce the production of the pro-inflammatory cytokines TNF, IL-1, IL-6 and IL-17 [27]. Therefore, the identification of pathways involved in “TNFR1 signalling”, “TNFR2 signalling”, “IL-1 signalling”, “IL-6 signalling” and “IL-17 signalling”, especially in the CD16^−^ monocytes from the SLE patients, supports the pathophysiological importance of IL-10 signalling events in SLE. Simultaneously, IL-10 signalling-mediated SOCS3 activation is known to suppress the production of the above-mentioned cytokines [28], and the dysregulated expression of SOCS in SLE was previously reported [29]. Accordingly, we detected the highly upregulated expression of *SOCS3* in all cell types from the SLE patients but not from the immunised ND. Another important function of SOCS3 is the regulation of CD4^+^ T cell differentiation [28], [30]. SOCS3 inhibits Th1 differentiation by binding to IL-12R to inhibit IL-12-mediated STAT4 activation, promoting Th2 differentiation. The Th2-skewed condition that is observed in the peripheral blood from patients with SLE may be caused by a mechanism that is controlled by SOCS3. This hypothesis is further supported by a previous mouse study showing that SOCS3-specific siRNA attenuates Th2 responses in vitro [31]. Therefore, the therapeutic modulation of SOCS3 expression is a promising treatment for SLE.

CD16^+^ monocytes are defined as migrated resident monocytes that are better inducers of pro-inflammatory cytokines, such as TNF and IL-1β, than CD16^−^ monocytes [32], [33]. To date, there are only two publications that compare the gene expression profiles between CD16^−^ monocytes and CD16^+^ monocytes in human ND [34], [35]. They found that CD16^+^ monocytes have more macrophage- and dendritic cell-like features with a superior ability for antigen presentation [33], [34], and CD16^+^ monocyte-derived macrophages have a higher phagocytic activity than CD16^−^ monocyte-derived macrophages [35]. However, our data did not support this observation at the transcriptional level. As long as we focus on the IFN signature genes, it is reasonable to assume that CD16^−^ monocytes from patients with SLE are responsible for eliciting inflammatory immune responses rather than CD16^+^ monocytes.

It is interesting that genes related to the JAK/STAT pathway were predominantly enriched in the CD16^+^ monocytes according to IPA. The JAK/STAT pathway is known to promote the expression of various IFN-inducible genes in the pathogenesis of SLE [36]. Both IL-10 signalling, which was top ranked in the CD16^−^ monocytes, and IL-9 and IL-15 signalling, which was top ranked in the CD16^+^ monocytes, use JAK/STAT signalling in their downstream transduction; however, the expression of *IL-10* and *IL-9* and their corresponding receptors was not significantly changed at the transcriptional level (data not shown). Only the expression of *IL-15* and *IL-15RA* was slightly upregulated in our data.

IL-15 plays a major role in the development of the inflammatory immune response, and the upregulation of IL-15 is involved in the development of several autoimmune and chronic inflammatory disorders, such as RA, psoriasis and celiac disease [37]. In the pathogenesis of RA, IL-15 stimulates T cells, induces TNF-α production by macrophages and supports the expansion and differentiation of Th17 cells to secrete IL-17 [37]. In patients with SLE, the serum levels of IL-15 were reported to be significantly increased [38]. Therefore, IL-15 signalling may be of major relevance in the pathogenesis of SLE. Alternatively, the upregulated expression of *IL2RG* may explain the significance of the IL-9 and IL-15 signalling detected in patients with SLE. The protein encoded by *IL2RG*, IL2Rγ, is an essential signalling component of many interleukin receptors, including those of IL-2, -4, -7, -9, -15 and -21, and is thus referred to as the common gamma chain [39]. The increased expression of the common gamma chain may change the response to these interleukins, subsequently upregulating the JAK/STAT signalling pathway.

In conclusion, this is the first study to demonstrate the cell-specific expression profiles of IFN signature genes in patients with SLE and ND immunised with YFV. The IFN signatures in autoimmunity and the normal immune response to pathogens are quite different with respect to the composition of the activated IFN-related genes and their expression levels. A unique signature of the dysregulated immune system in SLE may enable the further identification of relevant pathways in the pathogenesis of SLE and provide the basis for diagnostic tools or future therapeutic approaches. For example, “common” and “autoimmune-specific” IFN signature genes can be used as biomarkers for diagnostic purposes to differentiate between SLE flares and acute viral infections.

## Materials and Methods

### Patients and healthy subjects

For CD4^+^ T cells, six patients with SLE (average age: 29.0±7.6) and four ND (24.8±0.5) were recruited. For CD16^−^ monocytes, four patients with SLE (26.5±1.7) and four ND (24.8±0.5) were recruited. For CD16^+^ monocytes, four patients with SLE (26.5±1.7) and three ND (24.7±0.6) were recruited. All patients and ND were female. The same ND were examined before and after immunisation with YFV. The clinical characteristics of the SLE patients and information on the healthy donors are summarised in Table S7. Informed consent was obtained from all subjects, and the Ethics Committee of the Medical Faculty of Charité Universitätsmedizin Berlin approved the study.

For validation experiments we used public data on PBMCs of pediatric lupus patients (n = 10; GSE8650: http://www.ncbi.nlm.nih.gov/geo/query/acc.cgi?acc=GSE8650) [45]. These samples were hybridised on HG-U133A arrays.

### Immunisation of healthy donors with the yellow fever vaccine (YFV)

ND were immunised with a vaccine against the wild-type YF virus, which is a single-stranded RNA virus [40], without adjuvants. This vaccine consists of a live but attenuated strain of the yellow fever virus (YFV-17D). Based on its vaccination-associated clinical and serological manifestations, this immunisation can be regarded as a real viral infection, and gene expression analyses have revealed a type I IFN response under such conditions [13]. A total of 50 ml peripheral blood was taken 7 days after immunisation, when sufficient numbers of CD19^+^/CD27^++^ plasmablasts were detected by flow cytometry.

For validation experiments we used public data on PBMCs of yellow fever vaccinated individuals (d3, d7 and d21 after vaccination; n = 10; GSE13486: http://www.ncbi.nlm.nih.gov/geo/query/acc.cgi?acc=GSE13486) [46]. These samples were hybridised on HG-U133Plus 2.0 arrays.

### Sample processing and microarray

A total of 50 ml peripheral blood was collected in Vacutainer heparin tubes (Becton-Dickinson, Heidelberg, Germany), and erythrocytes were lysed in EL buffer (Qiagen, Hilden, Germany). Subsequently, granulocytes were depleted using CD15-conjugated microbeads (MACS, Miltenyi Biotec, Bergisch Gladbach, Germany). The CD15-depleted fraction was stained with a CD14-fluorescein isothiocyanate (FITC) antibody (Becton-Dickinson), a CD16-APC-Cy7 antibody (Becton-Dickinson), a CD3-Vioblue antibody (Becton-Dickinson) and a CD4-FITC antibody (Becton-Dickinson). Using a FACSAria cell sorter (Becton-Dickinson), CD4^+^ T cells, CD16^−^ monocytes and CD16^+^ monocytes were isolated with purities and viabilities of >97% [41]. After sorting, the cells were immediately lysed with RLT buffer (Qiagen) and frozen at −70°C. Total RNA was isolated using an RNeasy mini kit (Qiagen), and quality control was ensured by Bioanalyser measurements (Agilent Technologies, Santa Clara, CA, USA). The generation of cRNA, sample hybridisation using HG-U133 Plus 2.0 arrays (Affymetrix, Santa Clara, CA, USA) and scanning were performed according to the manufacturer's instructions.

### Microarray data analysis

The microarray data were analysed according to the following strategy: (1) primary data analyses for data normalisation and the generation of cell-files were performed using Affymetrix GCOS software; (2) the cell-files were imported into the BioRetis database (http://www.bioretis-analysis.de) [18], [42] to perform group-wise comparisons and filter the differentially expressed probe-sets, as described previously [43] (the detailed criteria used to select the significant probe-sets are described in Table S8); (3) the IFN-regulated transcripts were identified by comparing the differentially expressed probe-sets with a published list of 2442 IFN-related genes that were previously identified in PBMCs and monocytes [11], [17]; and (4) Genesis version 1.7.5 (http://genome.tugraz.at/genesisclient/genesisclient_description.shtml) was used to complete hierarchical cluster analyses.

The process of selecting lists of differentially probe is,based on 16 different groups of queries (columns in Suppl. Table 8) that have been combined by the Boolean operator OR. Parameters in each query group were combined by an AND-operation. In short the query parameters first select present genes with a signal threshold and at least one present call on at least one chip (>0%) of both groups. Selection of significant genes is subdivided into increased and decreased genes and both of them into a) the homogeneous group with at least 30% increase or decrease calls and a threshold of Bonferroni corrected t-test p-value and b) the group of heterogeneous genes with more than 50% increase or decrease calls. The t-test p-value in a) shows significance of the fold change (log2 FC) to be different to zero (null hypothesis is no change between both groups) so we can avoid selecting genes by FC. Query parameters implemented in the BioRetis-database were described in Menssen et al., 2009 [18] and were compared to well-known software tools like SAM (http://www-stat.stanford.edu~/tibs/SAM/) and dChip (https://sites.google.com/site/dchipsoft/) by using the Affymetrix Latin-Square dataset (http://www.affymetrix.com/support/technical/sample_data/datasets.affx).

The data discussed in this publication have been deposited in NCBI's Gene Expression Omnibus and are accessible through GEO Series accession number GSE51997 (http://www.ncbi.nlm.nih.gov/geo/query/acc.cgi?acc=GSE51997).

### Selection of 2442 IFN signature genes

A total of 2220 IFN-related genes were cited in a previous publication [17]. These genes were compiled from gene expression profiling studies that described the IFN signature in patients with SLE, from searches of the National Center for Biotechnology Information (NCBI) (http://www.ncbi.nlm.nih.gov/gene) and Ingenuity Pathway Analysis (IPA) (http://www.ingenuity.com) databases for genes with “IFN” in their gene or protein name or from an IPA search for direct regulators of the above genes to include upstream and downstream genes. In addition, we have included 222 IFN-related signature genes, which are specifically expressed in monocytes after in vitro stimulation for 90 min with IFN-α and in peripheral monocytes from patients with SLE [11].

### Functional annotation analysis

Biological function and canonical pathway analyses of the differentially expressed genes were performed using IPA software (Ingenuity Systems, Mountain View, CA, USA; http://www.ingenuity.com). IPA delivers a rapid assessment of the signalling pathways and biological processes that are most significantly perturbed in the gene expression data. For this analysis, we used the complete list of significantly differentially expressed IFN signature gene probe-sets without a cutoff of FC. Thus, 748 probe-sets in the CD4^+^ T cells from the SLE patients, 191 probe-sets in the CD4^+^ T cells from the immunised ND, 982 probe-sets in the CD16^−^ monocytes from the SLE patients, 540 probe-sets in the CD16^−^ monocytes from the immunised ND, 881 probe-sets in the CD16^+^ monocytes from the SLE patients and 542 probe-sets in the CD16^+^ monocytes from the immunised ND were applied (Table 1), and 554, 125, 686, 342, 629 and 356 genes, respectively, were used for evaluating the gene enrichment for known biological function and signalling pathways. A P value <0.05 (-log 1.25) was applied as the threshold for significance.

## Supporting Information

Figure S1 **In addition to** **figure 4** **, this supplementary figure demonstrates that the autoimmune-specific IFN signature of monocytes (figures a and b) and T helper lymphocytes (figures c and d) is not able to classify PBMC's from yellow fever vaccinated individuals 3 days (figures a and c) and 7 days (figures b and d) post vaccination.**
(TIF)

Figure S2 **Comparison of absolute expression magnitudes of “common” IFN signature probe-sets in SLE and immunized ND.** Fold changes (FC) of top candidate genes in CD4^+^ T cells, CD16^−^ monocytes and CD16^+^ monocytes from patients with SLE and immunized healthy donors (ND) with yellow fever vaccine (designated as “Viral infection”) are compared. (A) Comparisons of FCs for *IFI27*, *LY6E* and *IFI44L.* (B) Comparisons of average FCs considering total “common” IFN signature gene probe-sets.
(TIF)

Figure S3 **Comparison of gene enrichments in the selected canonical pathways in SLE and immunized healthy donors.** CD4^+^ T cells, CD16^−^ monocytes and CD16^+^ monocytes from patients with SLE and immunized healthy donors with yellow fever vaccine (designated as “Viral infection”) are analyzed by Ingenuity Pathway Analysis (IPA).
(TIF)

Table S1 **Differentially expressed IFN signature genes in CD4+ T cells (top candidate genes with cutoff of FC > = 2 or < = −2).**
(XLS)

Table S2 **Differentially expressed IFN signature genes in CD16- monocytes (top candidate genes with cutoff of FC > = 2 or < = −2).**
(XLS)

Table S3 **Differentially expressed IFN signature genes in CD16+ monocytes (top candidate genes with cutoff of FC> = 2 or < = −2).**
(XLS)

Table S4 **Comparison of selected genes for mean signal intensities and fold changes in different cell types from patients with SLE and immunized healthy donors by yellow fever vaccine.**
(XLS)

Table S5 **Comparison of biological functions by Ingenuity Pathways Analysis (IPA).**
(XLS)

Table S6 **Top ranked canonical pathways by Ingenuity Pathways Analysis (IPA).**
(XLS)

Table S7 **Clinical characteristics of patients with SLE and information of healthy donors.**
(XLS)

Table S8 **Selection criteria of significantly differentially expressed probe-sets by BioRetis database.**
(XLS)
