# Supplementary figures and images for: Cell-Specific Type I IFN Signatures in Autoimmunity and Viral Infection: What Makes the Difference?
Source: PLoS One. 2013 Dec 31;8(12):e83776. doi: 10.1371/journal.pone.0083776 (PMC3877094; doi:10.1371/journal.pone.0083776)

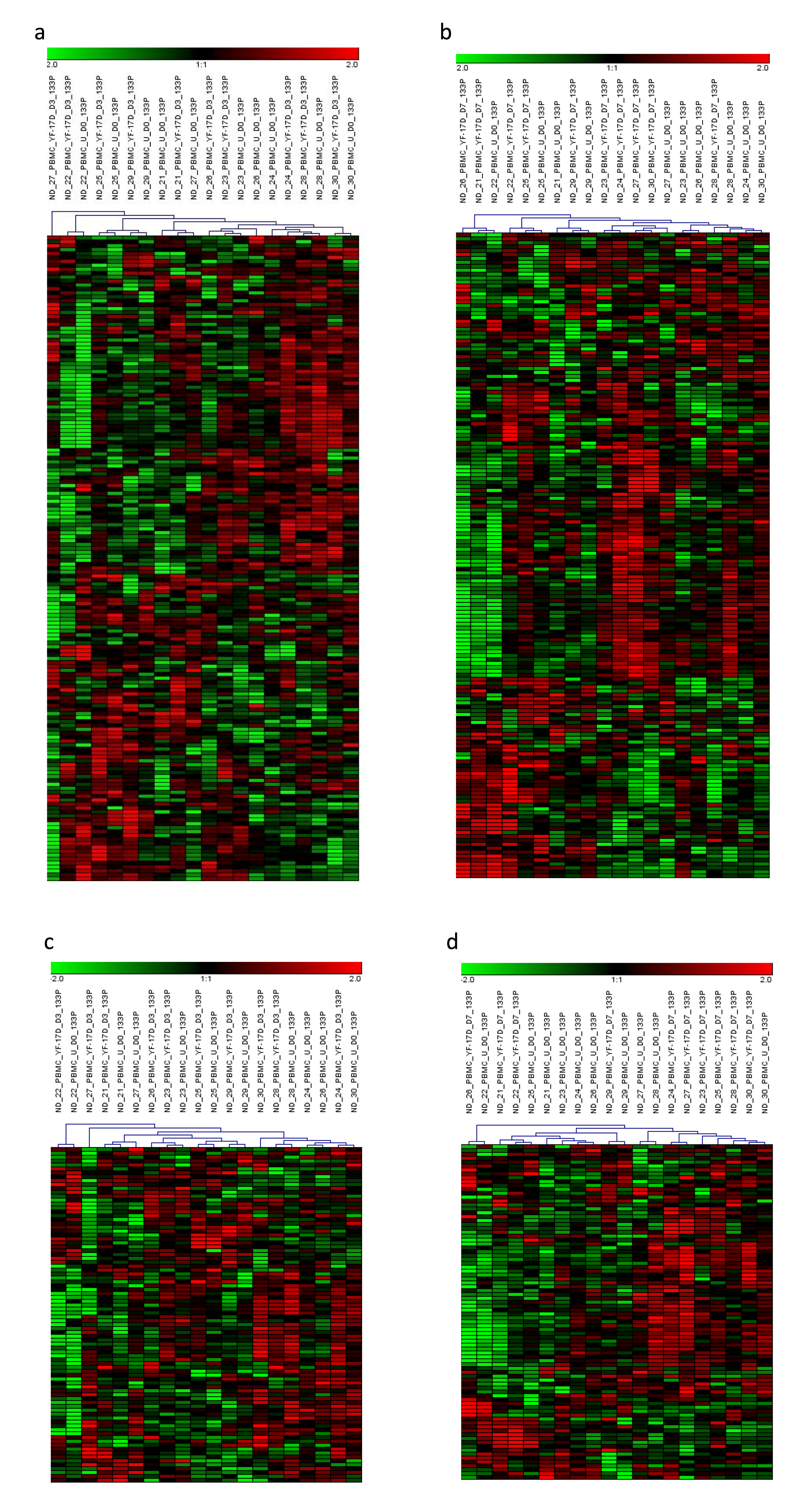

Supplement: Figure S1 — In addition to figure 4 , this supplementary figure demonstrates that the autoimmune-specific IFN signature of monocytes (figures a and b) and T helper lymphocytes (figures c and d) is not able to classify PBMC's from yellow fever vaccinated individuals 3 days (figures a and c) and 7 days (figures b and d) post vaccination. (TIF) [file pone.0083776.s001.tif]

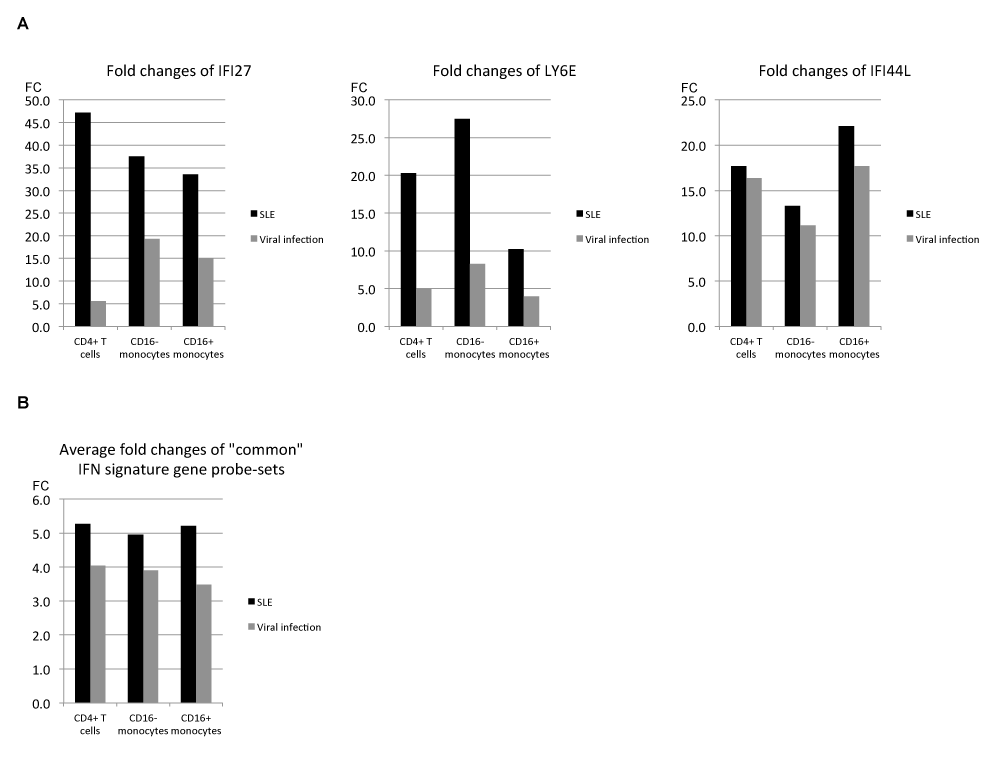

Supplement: Figure S2 — Comparison of absolute expression magnitudes of “common” IFN signature probe-sets in SLE and immunized ND. Fold changes (FC) of top candidate genes in CD4+ T cells, CD16− monocytes and CD16+ monocytes from patients with SLE and immunized healthy donors (ND) with yellow fever vaccine (designated as “Viral infection”) are compared. (A) Comparisons of FCs for IFI27, LY6E and IFI44L. (B) Comparisons of average FCs considering total “common” IFN signature gene probe-sets. (TIF) [file pone.0083776.s002.tif]

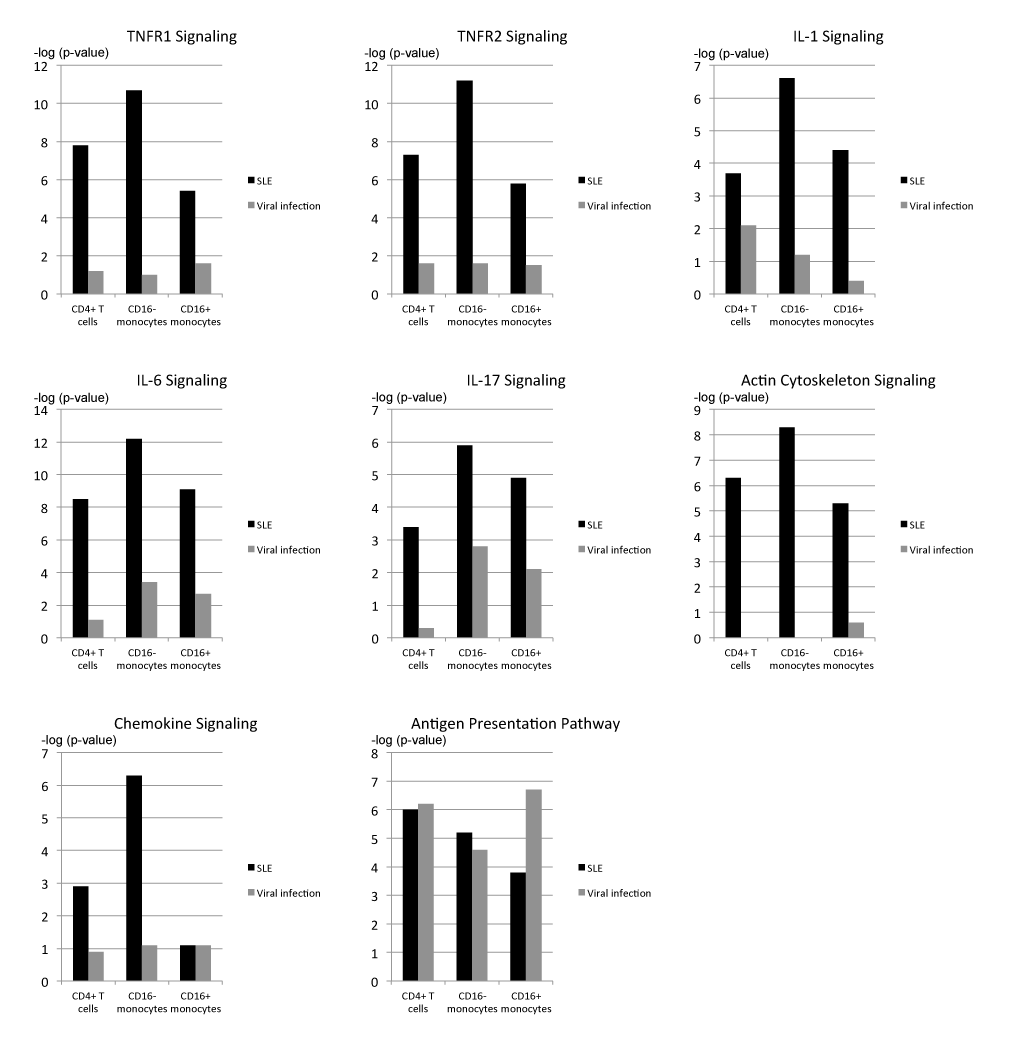

Supplement: Figure S3 — Comparison of gene enrichments in the selected canonical pathways in SLE and immunized healthy donors. CD4+ T cells, CD16− monocytes and CD16+ monocytes from patients with SLE and immunized healthy donors with yellow fever vaccine (designated as “Viral infection”) are analyzed by Ingenuity Pathway Analysis (IPA). (TIF) [file pone.0083776.s003.tif]
